# Pesticide Behavior in Soil Amended with Agricultural Waste and Agro-Industrial Byproducts: An Updated Review

**DOI:** 10.3390/jox16020046

**Published:** 2026-03-04

**Authors:** Gabriel Pérez-Lucas, Simón Navarro

**Affiliations:** Department of Agricultural Chemistry, Geology and Pedology, School of Chemistry, University of Murcia, Campus Universitario de Espinardo, E-30100 Murcia, Spain; gpl2@um.es

**Keywords:** pesticide residues, soil remediation, agricultural waste, agri-food byproducts, sustainable development

## Abstract

Farmers rely on pesticides to keep their crops safe from pests, diseases, and weeds. However, if pesticides are not used properly, they can have serious consequences for human and environmental health. Many pesticides are not easily biodegradable and persist in the environment for a long time. Their residues, including toxic metabolites, pose risks to non-target organisms, contaminate surface- and groundwater sources, and may affect future crops. Among other soil remediation actions, it is important to highlight the impact of agricultural waste and agro-industrial byproducts on the behavior of pesticides as a strategy to eliminate or at least minimize soil pollution by their residues. Waste from various food industries and agriculture poses a severe threat to the ecosystem and is difficult to manage properly. Agriculture and food production waste accounts for over 30% of total global agricultural output. Therefore, managing agri-food waste from different sources is crucial to promoting sustainable development with minimal environmental impact. Key components of waste management interventions in the agricultural circular and bioeconomy include incorporating crop residues and food waste into soils. For these reasons, we present an updated review of the impact of agricultural waste and agro-industrial byproducts on the behavior of pesticides in soil. The goal of this review is to promote the sustainable use of these wastes within the context of a circular economy.

## 1. Introduction

The world’s population is currently estimated to be 8.2 billion and is expected to grow over the upcoming decades. It will peak in the mid-2080s at approximately 10.3 billion, after which it will begin to decline, reaching 10.2 billion by the end of the century [1]. This is 6% (700 million), less than the projection from a decade ago. The circular sustainability model of traditional (subsistence) agriculture produces virtually no waste. Residues are recycled or used to maintain soil fertility. However, the increase in the global population has made it necessary to intensify agricultural production, adopt a linear agricultural model, globalize food distribution, and implement extensive storage and agro-industrial processing [2].

Population growth directly correlates with increased food demand and, consequently, increased food production. This increase is accompanied by large-scale food waste generation, including both edible and non-edible parts. The Food and Agriculture Organization of the United Nations (FAO) has stated that a third of all food produced around the world is either lost or wasted during the supply chain. The FAO generally identifies fruits, vegetables, roots and tubers as having the highest waste rates (up to 40–50%). The methodology for measuring food waste has evolved significantly in recent years. The United Nations now distinguishes between ‘food loss’, which occurs from production to retail, and ‘food waste’, which occurs from retail to consumer. According to recent reports, approximately 1.1 billion tons of food are wasted annually in the retail sector (12%), food service sector (28%), and households (60%) [3,4]. Recent data reveal that households in both upper- and lower-income countries waste a similar amount of food. It is estimated that food waste is responsible for 8–10% of global greenhouse gas (GHG) emissions (around 4.4 Gt CO_2_ eq per year), which is a significant contribution to the global climate crisis. The world’s third-largest emitter of GHGs would be food waste, and if it were a country, it would only be surpassed by China and the USA [5].

Food waste has economic, environmental, and social impacts. These impacts have arisen over time in both developed and developing countries, contributing to environmental and human deterioration, as little effort has been made to mitigate them. Nevertheless, the United Nations’ most prominent effort began in 2015 with the creation of the 2030 Agenda for Sustainable Development. This agenda sets out a 15-year plan to improve the global social, economic, and environmental situations, using Sustainable Development Goals (SDGs) as guidelines [6,7].

The primary distinction between waste and byproducts is their economic value and intended use or certainty of reuse. Both are secondary materials generated during a production process, but they are managed and regulated differently. According to Directive 2008/98/EC on waste [8], amended by Directive (EU) 2018/851 [9], a by-product is defined as a substance produced during a manufacturing process that can be used again following conventional industrial processing, provided that it does not have an adverse effect on human health or the environment. Conversely, waste is defined as any substance or object that is discarded by its holder, either temporarily or permanently. Waste is essentially unwanted or unusable material that does not add value to the main product or service. Essentially, the key difference amounts to usability and value: A byproduct is a sellable asset, whereas waste is a discardable liability.

Waste from various food industries and agriculture poses a severe threat to the ecosystem and is difficult to manage properly. Governments around the globe strictly advocate for the mitigation and valorization of agricultural waste. Therefore, managing agri-food waste from different sources is crucial to promoting sustainable development with minimal environmental impact. Appreciating waste by identifying fresh applications for it symbolizes a comprehensive and eco-friendly approach that prevents ecological pollution, delivers novel raw materials, and promotes zero waste by transforming waste into by-products. This aids in the execution of the circular economy strategy and the Waste Directive, which was endorsed by the Council of the European Union on 30 May 2018 [9]. Concerns about environmental stability and the depletion of natural resources have highlighted the importance of managing agro-industrial waste in innovative ways [10,11,12,13]. This waste was once considered a disposal problem but is now recognized as a valuable resource with great potential.

The circular bioeconomy promotes global sustainability through innovative waste management practices that support environmental goals and pave the way for a more sustainable future [14]. Increasing demand, population growth, urbanization, and rising incomes are revolutionizing the food, agricultural, and industrial sectors, leading to a significant increase in waste generation. Agriculture and food production waste accounts for over 30% of total global agricultural output. Agro-industrial waste contains valuable components such as fiber, vitamins, lipids, polyphenols, proteins, lignin, cellulose, and carbohydrates that can be used in various applications across different fields [15]. The circular economy replaces the ‘end-of-life’ model with the reduction, reuse, recycling, and recovery of waste materials. Traditionally, the 3Rs (Reduce, Reuse, and Recycle) were the pillars of waste management [16]. However, in the context of the circular economy, this model has become more ambitious, aiming to prevent waste from being created in the first place. Nowadays, advanced circular economy models talk about the 9Rs, adding six new key concepts for true sustainability such as the following: (i) Refuse: Choosing not to buy unsustainable products, (ii) Rethink: Using products more intensively (like carsharing), (iii) Repair: Fixing defective items so they keep functioning, (iv) Refurbish: Updating an old product (like furniture or machinery), (v) Remanufacture: Using parts of old products in new ones and (vi) Recover: Incinerating waste to generate energy (when it cannot be recycled).

This approach has a positive effect on the environment, the economy and society. It also encourages growth that can continue in the future. The term ‘bioeconomy’ refers to any sector that uses biological resources to produce food, feed, bio-based products, energy and services. In recent years, the circular economy and the bioeconomy have attracted global attention in policy, business and scientific research. These two sustainable models are becoming increasingly important [17]. Current models for managing agricultural residue and waste emphasize sustainability, profitability, technical feasibility, and potential for adoption. An integrated, sustainable, scalable, crop- and region-specific, socially inclusive, environmentally sound, and technically robust approach is taken by them.

Key components of waste management interventions in the agricultural circular and bioeconomy include the incorporation ‘in situ’ of crop residues, conservation agriculture, biofuel generation, biomass energy production, biochar production, residue composting, and biofertilizer production. These interventions also cover the production of different materials such as food coatings, packaging, biopesticides, animal and single-cell proteins, in addition to the extraction of bioactive molecules for use in a variety of applications.

On the other hand, agriculture heavily relies on plant protection products and other tools to protect plants and control pests, diseases, and weeds that reduce crop yield. In 2023, 3.8 million tons of active ingredients were used in agriculture worldwide for pesticides (plant protection products) [18]. On average, 2.4 kg per hectare of arable land was applied worldwide.

**Figure 1:**
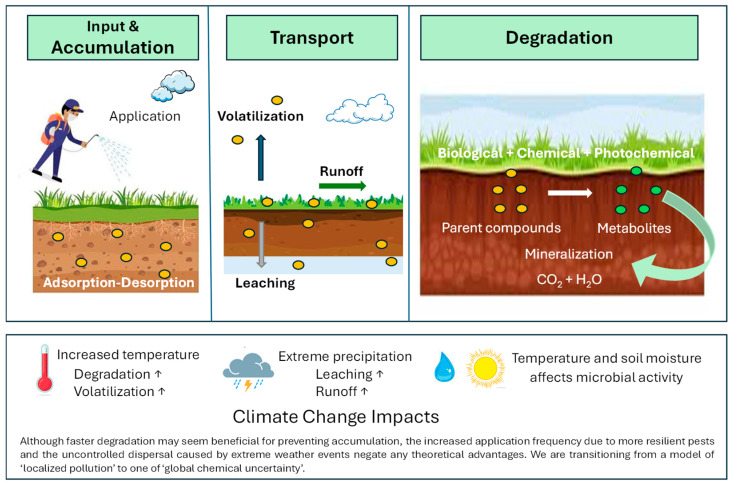
Behavior and fate of pesticides in the soil under climate change.

The behavior of pesticides in soil is primarily determined by seasonality, intensity, and increases in temperature and precipitation, as well as changes in land use. This indicates an indirect effect of the long-term impacts of climate change [19,20,21]. To protect, maintain, or restore the health of people, communities, and ecosystems, efforts should be made to recognize the relationship between pesticide use and climate, as this will help to inform future policy and practice using integrated and comprehensive approaches and partnerships. Figure 1 illustrates the behavior and fate of pesticides in soil under climate change scenarios.

Applying pesticides to cultivated soil is an effective way to control weeds, diseases, and pests, although this practice has raised environmental concerns because some pesticides are not easily biodegradable and persist in the environment for an extended period. Silva et al. [22] evaluated 76 pesticide residues in 317 samples of topsoil from 11 EU member states, examining six main cropping systems across the EU. The authors found that over 80% of the soils tested had residues of at least one pesticide, with a total of 166 different combinations identified. The compounds most commonly found in the soil samples, and at the highest concentrations, were glyphosate (herbicide) and its metabolite aminomethylphosphonic acid (AMPA), DDT and its metabolites, and some broad-spectrum fungicides such as epoxiconazole, tebuconazole, and boscalid. Another recent study found residues of 63 different pesticides in European soils, with one or more pesticides being detected in 70% of the sites investigated (croplands, grasslands and woodlands). Ten of the pesticides detected were phased out in the EU in 2018. The most prevalent types of detected pesticides were fungicides (54%), followed by herbicides (35%) and insecticides (11%). Researchers found the highest numbers of residues in croplands, followed by grasslands and woodlands [23]. The most frequently identified pesticides were also two herbicides (glyphosate and its by-product AMPA and pendimethalin) and two fungicides (boscalid and epoxiconazole). Furthermore, it was demonstrated that pesticide residues can have a number of effects on microbial functions, including the suppression of beneficial taxa such as bacterivore nematodes and arbuscular mycorrhizal fungi.

Double standards in pesticide trade pertain to high-income regions, primarily the EU and US, banning hazardous chemicals domestically due to health/environmental risks, while continuing to manufacture and export them to lower-income nations. The EU applies this precautionary principle and has some of the strictest legislation in the world. However, the situation is radically different on other continents. Many European agrochemical companies manufacture substances banned in Europe. Latin America has experienced the greatest growth in pesticide use, with nearly a 500% increase since 1990. Nearly 50% of the pesticides authorized in countries such as Argentina, Brazil, and Mexico are banned in the EU. Although Africa’s total volume is lower than that of the Americas, the risks are extremely high due to poor management, including precarious storage, a lack of protective equipment, and commercial pressure. Agrochemical manufacturers see Africa as an expanding market to compensate for restrictions in Europe. In Asia, countries such as Vietnam, Thailand, and India constantly have their exports rejected at European ports for exceeding maximum residue limits (MRLs) [24].

Residual pesticides can persist in the soil for periods ranging from a few hours to several years, impacting the environment and human life for extended periods of time. These residues, including toxic metabolites, pose risks to non-target organisms, contaminate water sources, and may affect future crops. In soil, pesticides undergo a complex process involving adsorption (binding), degradation (microbial, chemical, and photochemical), and transport [25]. Typically, less than 5% of pesticides act on the target pest. Pesticides primarily persist by binding to organic matter and clay, and microbial activity drives their breakdown. They mainly become mobile via leaching into groundwater or runoff, volatilization, and absorption (the uptake of pesticide molecules into plant tissues). These processes are influenced by soil texture, colloidal composition, pesticide properties and climatic conditions. Under specific conditions, field applications of certain pesticides can reach surface- and groundwater. This can happen via runoff and leaching [26,27,28]. The physicochemical characteristics of pesticides and soil properties such as texture, clay content, soil organic matter (SOM), and permeability, which all play a fundamental role in the leaching process, although the content of organic carbon (OC) is the most important factor inducing the pesticide adsorption and mobility in soil, and subsequently their disappearance [29]. A way to reduce pesticide leaching is the SOM content. The adsorption–desorption process principally regulates the rate and extent of biodegradation and pesticide leaching. Consequently, if a compound is adsorbed onto the clay–humic complex, it should not be affected by any other processes. Therefore, an effective strategy for reducing leaching is to increase the soil’s OM content through various agronomic practices, such as adding fresh or composted manure or plant biomass, because this increases the adsorption of non-ionic compounds. These practices, based on the use of modified or natural materials that have a high OC content, improve soil quality and reduce the amount of pesticides that leach through the soil [30]. As hydrophobic pesticides are attracted to these materials, OC is a significant factor in pesticide sorption [31], which typically renders the pesticides immobile in the soil, thereby improving their subsequent degradation and decreasing water pollution [32]. Accordingly, it is necessary to counteract the negative effects of long-term intensive agriculture on a soil’s physical, chemical and biological qualities by implementing sustainable agricultural practices, such as adding organic amendments (OAs), to the soil [17,33]. Various organic residues (ORs) have been investigated as potential sorbents for numerous pesticides in soil and water, mainly low-cost materials generated daily in large quantities from agricultural, urban, and industrial activities, such as sewage sludge, biosolids, and waste from agri-food industries [34,35]. Using low-cost adsorbents derived from agricultural waste provides a sustainable, economical alternative for treating wastewater and soil pollution caused by emerging pollutants [36]. These residues have been used in systems for treating agricultural effluents, including the biobeds implemented in many countries [37,38,39,40]. The EU recommends using biobeds for sustainable waste management [8]. Using biobeds can alter the final outcome of residues and prevent environmental problems caused by their accumulation. A biobed’s efficiency depends on the composition of the biomixture, which gives the system a high adsorption capacity and a microbial consortium capable of degrading xenobiotics, such as pesticides [41]. According to the aforementioned information, the objective of this updated review is to highlight the impact of agricultural waste and agro-industrial byproducts on the behavior of pesticides as a strategy for avoiding soil pollution from these xenobiotic compounds, based on research conducted over the past 15 years.

## 2. Searching Methodology

Throughout the selection process, the Science Citation Index Expanded (SCIE) of the Web of Science Core Collection (WoSCC) database was used to collect the data for this review. According to Birkle et al. [42], Web of Science (WoS) is the go-to resource for research evaluation and bibliometric analysis, recognized as the most comprehensive and widely used source. Additionally, it is also the oldest source of bibliographic data in the world. A variety of articles were located using different keywords, such as food waste, agro-industrial waste, biochar, biobeds, organic amendments, sustainable development, pesticide behavior, soil and circular economy, among others. These articles, including laboratory and field studies, were then selected according to the eligibility requirements (pesticide behavior in soils amended with agro-industrial waste) and screened using WoS filters. This study primarily used research from the last 15 years, starting from 2010, but due to their scientific importance, a few articles published before 2010 have been included. A total of 154 references were included in this updated review, excluding citations from international reference institutions. Eight of these references correspond to the period 2000–2009, and 146 correspond to the period 2010–2025. The present updated review has associated limitations as other bibliometric studies, including the database used, the Boolean strings chosen, the manual standardization, and the bibliometric parameters used to analyze the selected publications, among others. Extensive standardization was used to minimize the risk of bias during analysis. To ensure the rigor of the review, the following exclusion criteria were used: timeframe (publications prior to 2000), outdated legislation (studies based on regulations repealed), poor study design (studies that do not clearly explain their methodology), language (articles not available in English), and source types (editorials and letters to the editor).

## 3. The Role of Organic Waste as Soil Amendment

**Figure 2:**
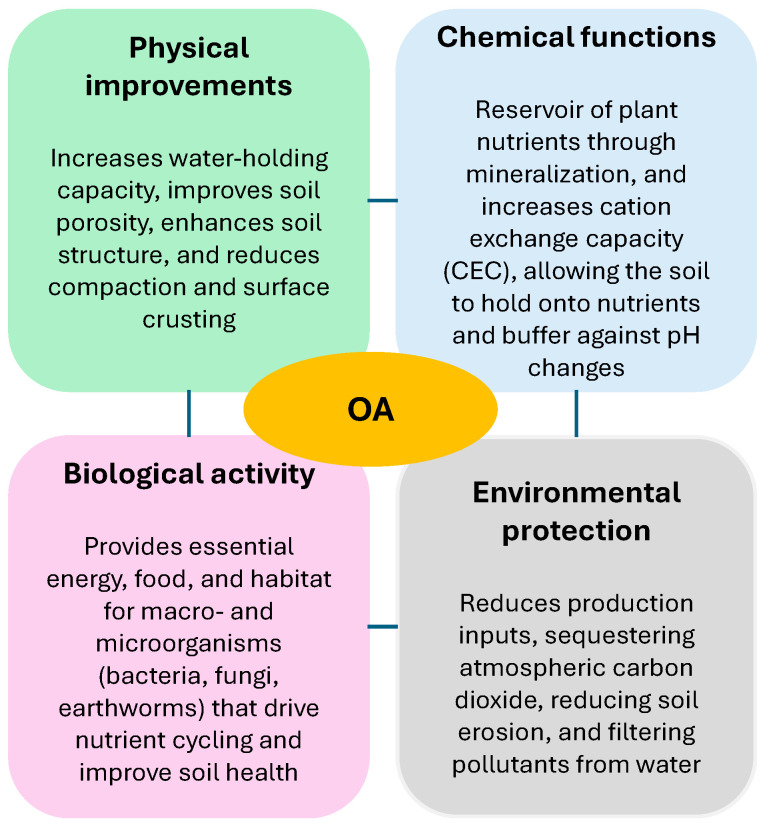
Functions of organic amendments (OAs) on soil properties.

Applying organic waste (OW) as soil amendments is a key part of current management practices for soils in arid and semi-arid regions. These practices aim to maintain proper structure and soil aggregate stability [43]. Using organic amendments (OAs) enables efficient recycling of OW resources and reduces reliance on chemical fertilizers [44]. Six types of organic amendments are commonly applied to soil: (i) animal manure, (ii) green manure and crop residues, (iii) food waste, (iv) manufacturing waste, (v) municipal biosolids, and (vi) composted sources. These amendments can replenish soil OM, improve soil physical (structure, porosity, bulk density, aeration, waterholding capacity, and mechanical resistance) and chemical properties (pH, organic matter content, and nutrient levels), reactivate microbial communities, and mitigate environmental pollution by xenobiotic compounds like pesticides (Figure 2). As a result, they allow for long-term increases in crop yield without damaging farmland, improving the quality and nutritional value of crops [45,46,47]. Conversely, OA can also have negative effects on the soil environment, such as heavy metal pollution and GHG emissions [44].

OA can accelerate biodegradation by stimulating microorganisms due to structural changes in soil porosity induced by a higher OC content. The addition of OW to soil enhances the presence of humified components, mainly humic and fulvic acids. These components are important for geochemical processes because they provide nutrients for plants and microorganisms. They also contribute to the acid-base buffering capacity of soils and improve soil structure, thereby improving moisture retention and aeration [48]. However, the role of various types of carbon found in OAs in the adsorption of organic pollutants remains unclear. The process of adsorption of pesticides by soil is increased by OA, but it is not possible to extrapolate results from one soil to another. This is because interactions between soils and OA can modify this process [49].

The fate of pesticides in the environment is often determined by adsorption, a process that plays a significant role in this regard [25]. The adsorption process between OA and pesticides involves three stages: (i) The adsorbate physically adsorbs onto the adsorbent’s surface, (ii) The adsorbate deposits onto the adsorbent’s surface, and (iii) The adsorbate condenses into the pore. The adsorption process comprises three distinct stages, each with its own unique characteristics. Firstly, no adsorption occurs (clean zone). Next, the mass transfer zone occurs and adsorption begins. Finally, equilibrium is achieved with the exhausted zone [50]. The underlying principles of adsorption are usually classified as either chemisorption, which is strong, or physisorption, which is weak, which are processes that take place at the interface of solid particles [51]. Weak hydrophobic interactions, van der Waals forces, hydrogen bonds, diffusion, and π–π bonds are the mechanisms through which sorption occurs [52]. These processes are crucially influenced by van der Waals forces and π–π electron donor–acceptor interactions. Many pesticides with aromatic rings exhibit π-electron systems. Therefore, the π–π electron donor–acceptor interaction can be recognized as the relationship between electron-deficient (acceptor) and electron-rich (donor) molecules. π-electron clouds interact through π–π stacking when pesticide molecules approach a material’s surface [53].

From an agronomic perspective, the addition of OW enhances biological activity and fertility. From an environmental perspective, the addition plays a significant role in the fate of several pollutants, such as aromatic hydrocarbons, heavy metals or pesticides. Furthermore, the addition of OA promotes the growth of fungi in the soil. The enzymes secreted by these fungi act non-selectively on pesticides and help with their initial conversion [54]. The resulting partially degraded pesticides (metabolites) are more amenable to bacterial mineralization. Adding OW increases the soil’s bacterial population, which often varies the rate and pathway of pesticide degradation depending on the amendment’s nature and its effect on the microbial community [55]. Fungi tend to attack most lignocellulosic organic materials. The process of degradation is accompanied by the release of extracellular enzymes, which act as catalysts for the bacterial breakdown of numerous pesticides [56]. The addition of OW enhances cometabolic pesticide biotransformation by expanding microbial activity [57,58,59,60,61].

Dissolved organic matter (DOM) exhibits similar characteristics to surfactants. These characteristics include lowering surface tension and increasing solubility, which makes compounds available to soil microorganisms. To stimulate microbial degradation during the bioremediation process, the soil can be amended with materials such as farmyard manure, biogas slurry, spent mushroom compost, coir pith compost, poultry litter, vermicompost, leaf compost, charcoal and wheat bran, among others. However, OA can have an inverse effect on degradation. Amendments can sometimes stimulate microbial activity without increasing herbicide degradation. This is probably because the specific microbial populations responsible for degrading pollutants are not stimulated. Sometimes, the addition of an auxiliary carbon source or OA decreases the pesticide degradation in soil because the auxiliary carbon source is more easily accessible than the toxic compound. Furthermore, the presence of OC from OW has been shown to enhance the adsorption of numerous pesticides to soil particles, thereby reducing their dissipation in amended soils [62] and acting as barriers to reduce pesticide leaching [32]. However, dissolved organic carbon (DOC) increases its dissipation and leaching, although this depends on the nature of the organic carbon [49]. Table 1 summarizes the main processes governing pesticide behavior and fate in the soil.

Herrero-Hernández et al. [63] studied the behavior of fluopyram and tebuconazole in two soils (silty and sandy loam texture) amended with spent mushroom substrate produced from cultivating *Agaricus bisporus* and composted with ophite at two application rates (5 and 20 g C kg^−1^) under field conditions. The adsorption distribution coefficients (*K*_d_) values for the adsorption of fluopyram and tebuconazole by unamended soils were between 0.45 and 1.39 mL g^−1^ and between 2.21 and 7.26 mL g^−1^, respectively. These values increased following the application of the amendments at both doses. *K*_d_ values indicate that tebuconazole is more strongly adsorbed than fluopyram by the unamended and amended soils due to its higher hydrophobicity. The authors found a significant correlation between *K*_d_ and organic carbon (OC) content for fluopyram (r = 0.92) and tebuconazole (r = 0.89) when considering both unamended and amended soils. The results suggest different dissipation mechanisms for both fungicides according to their dissipation half-lives (DT_50_): adsorption by soil OC prevented the dissipation of fluopyram, but facilitated the dissipation of tebuconazole, likely due to the formation of non-extractable residues. Other authors have noted that adding exogenous OM in the form of composted sheep manure increases the persistence of some fungicides (boscalid, myclobutanil, and penconazole) in amended soil. These fungicides showed a half-life (t_½_) greater than 110 days due to increased adsorption. Leaching experiments indicated that adding the composted material significantly limits the downward movement of fungicides through soil columns, considerably decreasing the amounts recovered in leachates [64].

## 4. Impact of Agro-Industrial Waste

Agro-industrial waste can be separated into three types: (i) recyclable and compostable waste, (ii) naturally occurring, non-recyclable, and non-compostable waste, and (iii) hazardous waste. Compostable waste is recyclable waste that can be reused on a farm or recycled at a recycling plant. This includes primary residues (straw, pruning, leaves, stover, stalks, bagasse, cobs, and animal dung or manure), as these arise directly from crop and animal production actions, as well as secondary residues (pits, shells, peels, husks, cakes, slurries, and slaughterhouse waste), as these arise from agro-allied industrial processing. Both types of residues are considered the least problematic to manage. Non-recyclable agro-industrial waste, such as plastic and metal containers and sheets, tires, shading or anti-stone netting, machinery, and metal structures for fences, covers, and irrigation facilities, results from farm construction operations and mechanization. These materials are usually bulky and difficult to reuse or recycle on the farm, making them the most challenging to manage. Finally, hazardous agro-industrial waste (agrochemicals, chemical containers, acids, wastewater, medicines, or detergents) poses critical immediate and long-term problems if not managed correctly. These wastes must be managed according to regulations established by the relevant authorities [17]. Only recyclable and compostable waste can be used as OA. Due to the loss of natural resources resulting from increased technological development, decisive and coordinated action is necessary to treat agri-food waste as a valuable resource that can be reused or recycled [65].

### 4.1. Agricultural and Agroforestry Waste

Agricultural waste refers to the residues generated during agricultural activities. This includes crop residues, such as the leftover parts of crops and fruit and vegetable waste, as well as animal waste, such as the droppings of livestock—mainly sheep, cows, pigs, and poultry. On the other hand, agroforestry is the practice of intentionally combining woody plants (such as trees or shrubs) with crop and/or livestock production systems in order to take advantage of the resulting ecological and economic interactions. Many non-renewable materials used in modern adsorbents come from agricultural waste. Consequently, the development of recyclable and renewable solid adsorbents tailored for agricultural applications has promoted sustainable development. A variety of agricultural solid waste has been transformed into high-value, renewable adsorbents [36]. These materials can be efficiently regenerated due to their physicochemical properties and used to remove pesticides and other emerging pollutants from wastewater and soil, thanks to their high adsorption efficiency [66].

Table 2 shows the main crop source materials studied for pesticide removal in soil, according to Liu et al. [66]. Grain crops are field crops whose harvestable parts are seeds, such as legumes, dry grains, such as cereals, or tubers. Cash crops are defined as farm products sold for profit. They are essential for global consumption and trade [67]. Cash crops are primarily classified into the following categories: fiber crops (e.g., cotton, hemp, and jute); oil crops (e.g., rapeseed, peanuts, and sunflowers); sugar crops (e.g., sugarcane and sugar beets); beverage crops (e.g., coffee and tea); and medicinal crops. Fruit peels and shells are an abundant waste product of fruit production. It is estimated that fruit peels account for 20–50% of the weight of processed fruits [68]. Disposing of fruit can impose significant environmental constraints and lead to severe pollution, so it is important to dispose of it properly. To address this issue, biotreatment of fruit peel and shell waste has been identified as an ecologically friendly remediation method for hazardous pollutants [69]. Animal feces are the primary form of livestock excretion and agricultural waste biomass [70]. A large quantity of feces can seriously harm the soil environment. Promoting the rapid development of livestock manure as a source of nutrients neutralizes it and encourages the production of organic fertilizers instead of chemical ones. Currently, there is growing interest in converting animal manure into BC due to its high ash content, which influences its interactions with pesticides [71].

Many studies have confirmed the adsorption ability of rice husk adsorbents for different pesticides such as fipronil, chlorpyriphos, α-and β-endosulfan, metolachlor, alachlor, p,p′-DDT, glyphosate, carbofuran, and diazinon, as reviewed by Liu et al. [66]. Moderate rice straw demonstrated improved sorption of imidacloprid, atrazine, and 13 other pesticides in an aqueous medium [72].

Besides being used as OA, spent mushroom substrate (SMS), an inexpensive and readily available in areas that produce mushrooms, could be used to control how pesticides behave when they are put in the soil. This approach is based on the ability of soils to control the adsorption/desorption of pesticides using SMS and the impact of these processes on the leaching and dissipation/biodegradation of these pesticides. The application of soil amended with composted or uncomposted SMS has been the focus of numerous studies in recent years due to its high organic matter (OM) content and low toxic element content, making it an attractive option for soil amendment. Different types of SMS have been assessed as soil OA, with variations depending on the components and the prior treatment. The application of these residues varies. Some are used immediately after being discarded from the production of mushrooms, while others are used after being composted under aerobic conditions. These conditions are designed to stabilize them. In addition to other uses, such as animal feed and energy feedstock, a source of lignocellulosic enzymes or dye decolorization, it is a useful material to use for the biodegradation of organic pollutants in soils, including pesticides [73].

The retention of many pesticides, primarily fungicides, in SMS-amended soils has been reported [74]. The SMS amendment rate, SMS type (composted or fresh), and SMS-soil incubation time were the factors examined in these studies. The goal was to analyze how these variables affect pesticide adsorption/desorption when SMS is applied to soil. Marín-Benito et al. [75] assessed the capacity of SMS to adsorb pesticides. They examined the adsorption/desorption capacity of seven fungicides (benalaxyl, metalaxyl, tebuconazole, penconazole, cyprodinil, pyrimethanil, and azoxystrobin) in SMSs produced from three different types of mushrooms: *Agaricus bisporus*, *Pleurotus* spp., and *Lentinula edodes* (also known as shiitake). The results show that the three SMSs have a high level of efficiency in adsorbing the most hydrophobic compounds (penconazole, tebuconazole and cyprodinil), with the remaining percentage of the fungicide adsorbed by the SMSs varying from 20% to 80% for tebuconazole and cyprodinil, respectively. Consequently, various studies have been conducted to compare the changes in pesticide retention in soils that have been untreated (unamended) and soils that have been treated with SMS. For instance, Marín-Benito et al. [76] examined how adding fresh and composted SMS from *Agaricus bisporus* cultivation affected the adsorption of penconazole and metalaxyl. They reported that the SMS-amended soils had a higher adsorption of both fungicides. However, the fresh SMS had a higher adsorption capacity of penconazole than the composted SMS due to its higher OC content. This was not the case for metalaxyl, as there were no noteworthy differences between the two materials. Table 3 summarizes the effects of different OAs on the adsorption, degradation and leaching of some pesticides in soils.

To prevent and/or control the contamination of surface and groundwater by pesticides, Álvarez-Martín et al. [77] tested the use of *Agaricus bisporus* as a biosorbent applied at various rates (2–75% *w*/*w*) to soils with different levels of nonpolar (triadimenol and tebuconazole) and polar (pirimicarb and cymoxanil) pesticides. They observed an increased adsorption of up to three times for all pesticides in amended soils at SMS application rates between 2 and 10%, and an increase of up to 20 times at application rates between 25 and 75%.

Applying SMS with high levels of OC and DOC to agricultural soils could alter the behavior of pesticides and alter how soils retain or transport them to surface and groundwater. Depending on its nature and the amount applied, these processes may be affected when SMS is used as a soil amendment. Studies on the mobility of pesticides in SMS-amended soils (*Agaricus bisporus*) have been reported for compounds with different properties, such as penconazole and metalaxyl [76]. Using SMS may prevent groundwater pollution from metalaxyl due to its higher adsorption and lower leaching. In contrast, penconazole did not leach after the experiment and was immobilized in the soil because of its high adsorption coefficient. As reviewed by Marín-Benito et al. [74], the outcomes of trials performed in both field and lab settings suggest that SMS has an impact on the dissipation and bioavailability of pesticides with varying properties in soils amended with SMS.

Grenni et al. [78] studied the degradation of terbuthylazine with the addition of wood amendments, such as pine and oak. Pine residues increased terbuthylazine sorption to soil and hindered microbial degradation due to their high sorption capacity, which decreased the herbicide’s bioavailability. Conversely, herbicide sorption did not significantly increase in the presence of oak residues. Another study found that the degradation of metalaxyl and alachlor followed this order: pine-amended soil < oak-amended soil < non-amended soil [79]. Other results reveal that the efficacy of these reactive wood barriers in decreasing the leaching of the pesticides linuron, alachlor, and metalaxyl (which have contrasting physicochemical characteristics) depends on the type of wood waste, the hydrophobicity of the pesticide, and the water flow rate adopted [80]. Similarly, Marín-Benito et al. [32] observed a decrease in the leaching of ethofumesate and terbutryn in soil when pine waste was used as an organic barrier.

**Table 3:** Effects of OAs on the adsorption, degradation and leaching of some pesticides in soils.

Pesticides [Reference]	Soil	OA	Main Findings
Chlorpyrifos, endosulfan sulfate, chlorfenvinphos, lindane, alachlor, and atrazine [81]	Silty clay loam	Composted sewage sludge mixed with pruning residues, orujillo, and chicken manure	The application of three different concentrations (2, 5 and 10% *w*/*w*) of OAs to the soil resulted in increased sorption (*K*_f_ value) of the six pesticides, which was higher for two of the more hydrophobic compounds (chlorpyrifos and endosulfan sulfate) (Log *K_ow_* 4.7 and 4.8, respectively) and lower for the more polar ones (atrazine and alachlor) (Log *K_ow_* 2.7 and 3.1, respectively).
Azoxystrobin, metalaxyl, penconazole, pyrimethanil, iprovalicarb, benalaxyl, tebuconazole, and cyprodinil [82]		Composted *Agaricus bisporus*, fresh *Agaricus bisporus*, fresh *Pleurotus* spp., and fresh *Lentinula edodes*	Adsorption: *K*_f_ (13.0–1385) and *K*_d_ (9.65–698). Composted *Agaricus bisporus* was the most effective for retention of all the fungicides, while fresh *Agaricus bisporus* was only for the most hydrophobic (penconazole and tebuconazole).
Linuron, diazinon, and myclobutanil [83]	Sandy clay loam and sandy loam	Sewage sludge (SS), grape marc (GM), and spent mushroom substrate (SMS)	Adsorption was not related to pesticide hydrophobicity. For unamended soils, distribution coefficients (*K*_d_) ranged between 1.77 and 6.60 mL g^−1^ for linuron, 0.54–5.52 mL g^−1^ for diazinon, and 1.35–4.52 mL g^−1^ for myclobutanil, and increased significantly for amended soils: up to 4.8 times for linuron, 6.9 times for diazinon, and 5.3 times for myclobutanil. *K*_d_ values revealed the highest adsorption of linuron and diazinon by GM and of myclobutanil by SMS.
Terbuthylazine [78]	Clay loam soil	Pine and oak residues	Pine residues increased the sorption of terbuthylazine to soil, while in the presence of oak residues, the herbicide sorption did not increase. Two months later, 73% of the herbicide applied still persisted in the pine-amended one and 63% in the oak-amended and unamended ones.
Metalaxyl and alachlor [79]	Sandy loam	Pine and oak sawdust	The degradation rate for both pesticides followed the order: pine amended soil < oak amended soil < non-amended soil. The faster degradation rate in non-amended soil was attributed to the higher sorption of pesticides by wood-amended soils.
Azoxystrobin, cyprodinil, fludioxonil, hexaconazole, kresoxim-methyl, pyrimethanil, tebuconazole, triadimenol, pirimicarb, and propyzamide [84]	Clay loam	Composted sheep manure and spent coffee grounds	The addition of OAs drastically reduced the movement of the studied pesticides. Only two pesticides were found in leachates from amended soils: pyrimethanil (<1%) for both, and pirimicarb (44%) in the soil amended with spent coffee grounds. A decrease in pesticide leaching was observed with the increase in dissolved organic matter (DOM) of leachates.
Metobromuron and chlorbromuron [85]	Silty clay loam	Orange peel, beer bagasse, grape pomace, and gazpacho waste	The sorption coefficients (*K*_OC_) increased in the amended soils. Metobromuron was found in leachates in all cases, although a marked reduction was observed in amended soils, while chlorbromuron was mainly retained in the topsoil layer. The disappearance time (DT_50_) for metobromuron and chlorbromuron in soil ranged from 11 to 56 d and 18 to 95 d, respectively.

Additionally, Lorenzo et al. [86] demonstrated the effectiveness of various organic waste materials, such as *Vicia faba* pods, *Urtica dioica* residues, spent coffee grounds, and corn cobs, as biostimulants and bioherbicides under lab and field conditions. Pot assays revealed that *Vicia faba* pods, *Urtica dioica* residues, spent coffee grounds, and corn cob waste exhibited the most potent inhibitory effect. During periods of scarce rainfall and warm days, the use of spent coffee grounds in the field led to a reduction in the biomass of naturally emerged weeds and an increase in crop growth.

### 4.2. Agri-Food Processing Waste

Agro-industrial processing waste includes peels and seeds from processed fruits and vegetables, grain milling waste, and oilseed processing waste, among others. The processing of fruits and vegetables generates the highest volume of solid waste. This waste often consists of non-edible or ‘unattractive’ parts of produce, such as pomace, which is the pulpy residue remaining after fruits like apples, grapes, and tomatoes are crushed for juice or oil. Other waste includes peels and skins, such as potato skins, citrus peels, and onion skins; seeds and husks, such as grape seeds and cocoa bean shells; and cullage, which is whole fruits or vegetables discarded because they do not meet retail standards for size, shape, or color. On the other hand, processing grains into flour or polished rice leaves behind fibrous outer layers, including bran (the hard outer layer of cereal grains, such as wheat, rice, and oats); straw and stover (the stalks and leaves left in the field or separated during initial processing); rice husks (the indigestible skin of rice kernels, often used for biomass energy); and brewers’ spent grain (the solid residue left after the brewing industry extracts the wort from malted barley). Additionally, when oil is extracted from plants, the ‘leftovers’ are often highly nutritious, such as with oil cakes, as with the solid residue remaining after pressing soybeans, sunflowers, rapeseeds, or olive mill wastewater—a dark polyphenolic liquid generated during olive oil production [11].

The use of grape marc, which comprises grape stalks, seeds, and skins left after the crushing, draining, and pressing stages in wine production, as OAs reduces the disappearance time for three pesticides such as linuron, diazinon and myclobutanil [87]. Pérez-Lucas et al. [85] evaluated the adsorption, leaching, and dissipation processes of metobromuron and chlorbromuron, phenylurea herbicides, in a silty clay loam soil. They examined both unamended and amended soil with various agro-industrial waste products, including brewer’s spent grains, orange peel, grape pomace, and gazpacho waste. The researchers observed an increase in sorption coefficients (*K*_OC_) in the amended soils. Metobromuron was detected in all leachates, although amended soils exhibited significantly lower concentrations. Conversely, chlorbromuron was predominantly retained in the soil, particularly in the top layer.

## 5. Technological Approaches

In recent years, several technologies for modifying agri-food waste have been proposed. The main methods include converting waste into biochar, stabilizing waste through composting, and using biobeds made from different biomixtures.

### 5.1. Biochar

**Figure 3:**
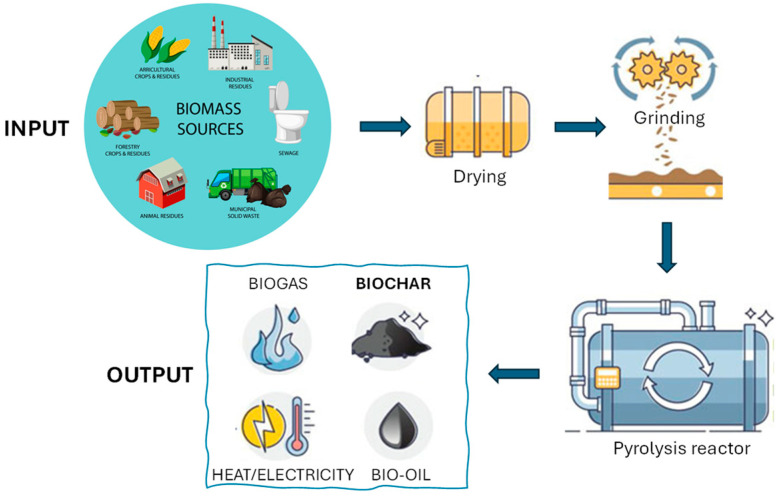
Schematic of the pyrolysis process used to obtain biochar.

Biochar (BC), a carbonaceous material, is produced by pyrolyzing biomass residues under oxygen-limiting conditions [88]. Pyrolysis is the most cost-effective and efficient method of producing the desired result among other various thermal conversion techniques, such as torrefaction, gasification, and hydrothermal carbonization, in which biomass is heated at relatively low temperatures (300–900 °C) in an oxygen-free environment. This technique can be subdivided into two major types: slow and fast pyrolysis, which differ in their heating rates [89]. In the process of pyrolysis, liquid, solid, and gaseous products are generated (Figure 3).

The term ‘biochar’, a carbon-rich material, refers to a solid product that is similar to charcoal. The liquid products consist of a non-aqueous phase (called bio-oil) and the aqueous phase (known as aqueous pyrolysis liquid). A mixture of H_2_, CH_4_, CO, and CO_2_ is produced in addition to low concentrations of other compounds, such as ethane and propane. BC production generally uses waste biomass, including. aqueous biomass, wood waste, crop residues, food processing waste, animal manure, paper industry waste, sewage sludge and others [90]. BC derived from plant biomass is obtained from sources such as wheat straw, corn cobs, and rice husks, among others. This material has a high pore capacity, large specific surface area, tunable surface functional groups and good environmental compatibility. These properties make BC an inexpensive and efficient adsorbent. Consequently, greater emphasis is required on the search and use of BC derived from plants for the purpose of pesticide removal [33]. Using biomass to produce BC for soil amendment provides a solution that benefits both soil remediation and agricultural waste utilization [91,92].

A high water retention capacity is an important feature of BC, a porous material. The improvement of aeration conditions and the provision of suitable conditions for microbial growth are both facilitated by this capacity [93]. This, in turn, reduces metabolic activity in microorganisms and increases the breakdown of pesticides [94]. BC changes soil properties, increasing microbial biomass, enzyme activity, and microbial community structure [95]. According to studies by Sohi et al. [96] and Harter et al. [97], water-soluble components, such as carbohydrates, acids, aldehydes, alcohols, and ketones, may positively impact the soil microbial community. These components are easily metabolized by soil microorganisms. Conversely, the presence of toxic substances such as polycyclic aromatic hydrocarbons, acrolein, formaldehyde, cresols, and other carbonyl compounds may have fungicidal or bactericidal activity, depending on pyrolysis conditions.

BC, when used as an OA, has been shown to enhance soil fertility, boost crop yields, decrease carbon emissions, and revitalize degraded soil [93,94]. The pore size and surface area of the BC produced are affected by the pyrolysis temperature and feedstock. BC obtained at high temperatures (600–700 °C) generally has a higher surface area than BC produced at low temperatures (<400 °C) [86]. In agriculture, a high BC surface area is important because it increases the water retention capacity of soil with large macropores when applied to it.

The impact of BC on polluted soils depends on diverse factors, such as the pyrolysis process, the physicochemical properties of BC, the application method, and the soil conditions [98]. These factors can lead to substantial discrepancies in the removal or reduction of pesticides. Adding BC to soil reduces pesticide soil pollution because of its high binding capacity for pesticides. Adding BC increased the soil’s adsorption capacity for pesticides and boosted the diversity and abundance of soil microbial populations. Pesticides are adsorbed on the surface of BC through physical and chemical adsorption during pesticide adsorption. This process does not involve chemical reactions, thereby demonstrating the material’s stability [35]. BC application also reduces pesticide uptake by plants, which is probably associated with the high porosity of BC [81]. BC plays a crucial role in various soil processes, including adsorption, desorption, biodegradation and leaching [52]. Adding BC to a pesticide-polluted environment offers several advantages: (i) it increases the soil’s water-holding capacity; (ii) it improves the soil’s aeration environment; and (iii) it provides a habitat for microorganisms to grow, facilitating the metabolic activities and pesticide degradation of the microbial community [99]. BC has been reported as the preferred compound for reducing the bioavailability of pesticides in soil environments. It also increases soil fertility and mitigates climate change. As an eco-friendly soil remediation material, BC can efficiently immobilize pesticides [52]. BC could be an effective adsorbent due to the following reasons: (i) it is obtained with a low carbon footprint, (ii) it is highly favorable for waste treatment, and (iii) it minimizes environmental damage by reducing GHG effects. BC has been reported to substantially reduce soil pesticide levels by sequestering and adsorbing pesticides or their derivatives in both laboratory and field studies [91,100,101,102,103,104,105,106,107,108,109,110]. Table 4 shows the effect of different BC on the decrease in pesticides in soils. The presence of BC in the environment generally leads to a decrease in the risk of human exposure to pesticides due to the decreased dissipation and increased sorption.

BC significantly affects the environmental behavior and fate of pesticides in soil. Due to its large surface area and high porosity, BC reduces the bioavailability of pesticides to soil microorganisms, thereby slowing biodegradation rates. On the other hand, BC has been shown to stimulate microbial activity by offering accessible carbon and additional nutrients, thereby enhancing the rate of pesticide biodegradation in soil [94]. The effect of BC on the behavior and fate of pesticides in soil depends on its properties. These factors are influenced by the feedstock and pyrolysis process used to produce the BC, which is regarded as an efficient solution for addressing waste originating from plants or animals, with the aim of reducing environmental contamination [105]. The stability of BC in soil is a key factor in determining its effectiveness for pesticide remediation. The potential of BC in the bioremediation of pollutants can be studied using the H/C and O/C molar ratios as important indicators, in addition to pyrolysis conditions and the type of raw material [111].

New studies and reviews are frequently published in the BC research field, which is currently very productive. Both low and high pyrolysis temperatures have negative and positive effects on pesticide sorption in soil. Low temperatures increase the number of functional groups, while high temperatures increase surface area. Therefore, it is necessary to have a clear knowledge of these effects and mechanisms in order to engineer BC generation with desired properties. Some authors have pointed out that BC produced from wood pellets, when added to soils result in almost complete sorption of two herbicides (aminocyclopyrachlor and bentazone) [112]. However, BC made from macadamia nut shells reduced the absorption of both herbicides in soils compared to unamended soil. The authors attributed this fact to the herbicides competing with the DOC from the BC for sorption sites.

Over the past few years, various authors have conducted in-depth reviews of BC’s role in the processes of sorption, desorption, degradation, and leaching of pesticides in soil under laboratory and field conditions. These reviews have also explored the key characteristics of BC, such as porosity, surface area, pH, carbon content, surface functional groups, aromatic structure, and mineralogical composition [53,99,104,106,110,113,114,115,116,117].

Ćwieląg-Piasecka et al. [118] demonstrated that BC produced from wheat straw specially attracts non-ionic pesticides with relatively high log *K*_OW_ values due to its relatively hydrophobic character. The main mechanism postulated for their attraction to BC is hydrophobic bonding. In addition to the structural properties of the sorbent, pH is the main factor that governs the sorption equilibrium of the mixtures. The sorption of atrazine and simazine (triazine compounds) by BC was favored by low pH. When both compounds co-existed, competitive sorption occurred between the two pesticides, which resulted in a decrease in sorption capacity [119]. Cwielag-Piasecka et al. [118] studied the retention of five pesticides (2,4-D, MCPA, metolachlor, carbaryl, and carbofuran) on de-ashed wheat straw BC under laboratory conditions. They demonstrated that hydrophobic pesticides, such as metolachlor and carbamates, exhibited comparably high and irreversible adsorption on BC due to pore filling. In contrast, the hydrophilic and ionic phenoxyacetic acids were weakly and reversibly sorbed. BC has been proven to be the most effective at increasing the sorption capacity of pesticides in soil compared to other OA, such as straw or compost [120]. BC amendments enhance the activity of bacteria that degrade difenoconazole by modifying the chemical properties of the soil. This eventually reduces the bioavailability of difenoconazole in polluted soils [121].

According to Cederlund et al. [122], the leaching through laboratory columns of two herbicides (MCPA and diuron) was reduced by the application of a wood-based BC, with retention being directly related to the thickness of the BC layers, although the addition of BC did not affect the leaching of chlorpyrifos or glyphosate. The addition of pesticides to the BC, which was then added to the soil column, decreased leaching. This suggests that using the BC as an adsorptive layer on or close to the soil surface may be a viable strategy for mitigating pesticide leaching. This would be particularly helpful in places where pesticides are frequently used and might be spilled. The adsorptive behavior and mechanism of grape pomace-derived BC in removing cymoxanil were studied by other authors [123]. The results indicated that the adsorptive mechanisms were chemical sorption and multilayer formation on the heterogeneous surface of BC. Losacco et al. [109] noted that the binding of BC with pesticides depends on its structure and nature. They found that adding wood BC significantly reduced the leaching of azoxystrobin compared to unamended soil. However, a mixture of BC made from agricultural waste of various origins, such as agricultural waste, pomace, olive pomace bran, fruit shells, hazelnuts, and wood processing waste, was more effective at mitigating spinosad.

Recently, efforts have been made to improve the physicochemical and adsorption properties of BC by creating modified BC [124]. The goal is to expand its applications. The following aspects are of the utmost importance: (i) the sources and modification methods of BC for pesticide remediation, (ii) the effect of BC on the fate of pesticides during remediation, (iii) the effect of BC on pesticide-polluted soils, and (iv) the potential issues associated with the large-scale implementation of BC for pesticide remediation.

### 5.2. Composted Waste

**Figure 4:**
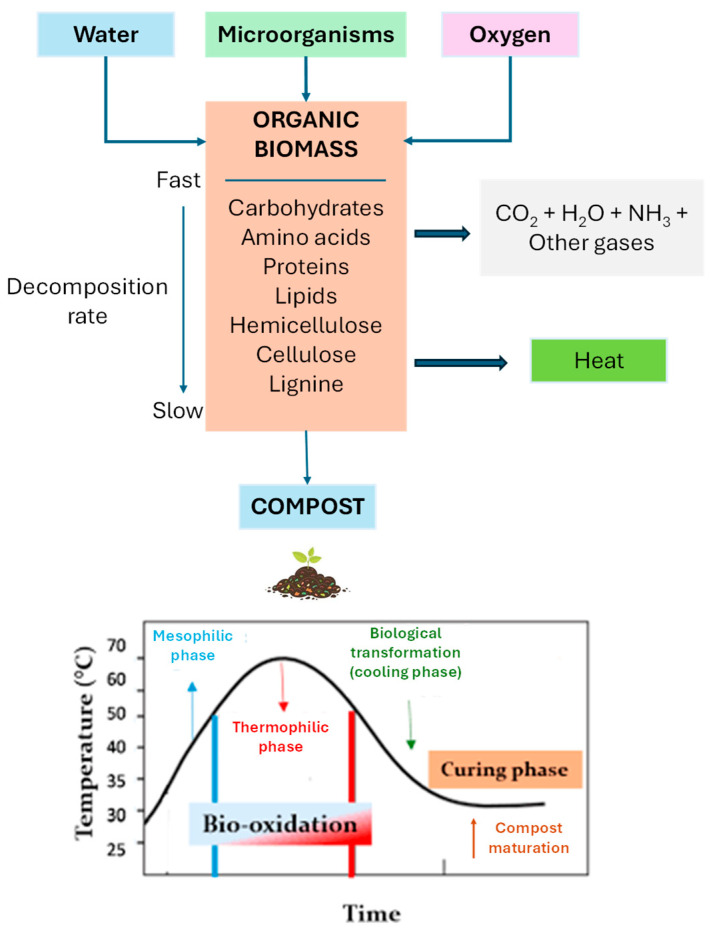
Scheme for the composting process.

Incorrect waste management is widespread and can be dangerous. It can be substituted with a safer method, such as composting: the controlled conversion of biodegradable organic materials and waste products into stable materials with the help of microorganisms [125]. Composting is a fundamental agricultural process that helps to recycle farm waste. Compost helps to improve soil fertility and plant yield. In addition, composting helps protect groundwater from pollution, unlike landfilling, which can threaten groundwater quality. This is because composting reduces the number of microbes and chemical pollutants. Composting is a process that has many benefits for agriculture. It increases productivity and improves the quality of soil by adding nutrients and beneficial organisms [126]. Additionally, soil polluted with pesticides and other xenobiotics can be remediated using compost, as this can reduce the toxicity of certain chemical pollutants by absorbing or breaking them down [127]. This, in turn, helps to ensure food security. Two further areas that need to be addressed are temperature regulation and the control of oxygen flow, in addition to humidity. These are key to the microorganisms carrying out the composting process. The different microorganisms at each stage function at specific temperatures, so these must be carefully monitored. They also require oxygen to reduce the activity of anaerobic bacteria because increased anaerobic activity increases the production of CO_2_ and the release of H_2_S, which can cause health issues. Figure 4 shows a schematic drawing of the composting process.

In general, the quality of mature compost largely depends on the type of feedstock material used and the degree of decomposition. Household organic waste, such as separately collected biowaste, as well as green waste, such as grass clippings from the garden, may contain non-decomposable materials, like plastic debris, rendering it unsuitable for use on soils. On the other hand, composted sewage sludge and manure-based composts often have high salt content and may contain chemicals and heavy metals. However, materials derived from animal waste have been found to be more effective than plant residues in building SOM [128]. Therefore, the quality of compost should always be monitored before application to the field.

As the material becomes more biologically stable during composting, compost can contribute to the humic matter in soil. Generally, the humic content of soil increases by up to 2% with repeated additions of compost, which has positive effects on physical properties and soil structure. Generally, bulk density and erosion decrease, while pore volume, aggregate solidity and stability, and amount of macro- and mesopores, as well as water infiltration, increase after compost application [120].

Marín-Benito et al. [82] reported on the adsorption–desorption capacity of various fungicides by SMS produced from three types of mushrooms (three fresh substrates from *Pleurotus* spp., *Agaricus bisporus*, and shiitake, as well as composted *A. bisporus*), showing that the SMS from the composted *Agaricus bisporus* had the highest adsorption capacity in all cases. Rodríguez-Cruz et al. [83] studied the effect of composted SMS (25% *Pleurotus* spp., 75% *Agaricus bisporus*) on the adsorption of diazinon, linuron, and myclobutanil in three different soils. The application of the amendments resulted in an increase in the adsorption capacity of the pesticides in all cases, according to the results. Herrero-Hernández et al. [129] used the same composted SMS to evaluate changes in fungicide adsorption with SMS + soil incubation time at the field scale. The authors observed that azoxystrobin adsorption was higher in soils amended with SMS, which is consistent with the increase in OC content after SMS was added. However, the adsorption of azoxystrobin decreased in amended soil over time. This has been linked to changes in OC content, as reported for other fungicides in SMS-amended soils [82]. In contrast, Herrero-Hernández et al. [130] observed that adding composted SMS (75% *Agaricus bisporus* and 25% *Pleurotus* spp.) increased tebuconazole adsorption, both immediately and over time, despite a decrease in OC content in amended soils. This is likely due to a decrease in DOC content over time, which would explain the observed results.

The impact of two OAs, agro-industrial waste (spent coffee grounds) and animal manure (composted sheep manure), on the mobility of pesticides frequently used to protect peppers in the field was studied by Fenoll et al. [84]. The movement of the pesticides was drastically reduced by the addition of these amendments. Pyrimethanil and pirimicarb were the only two pesticides detected in leachates from amended soils. Pyrimethanil was found in trace amounts (less than 1%) in both soils, while pirimicarb was detected at a concentration of 44% in soil amended with spent coffee grounds. Decreases in pesticide leaching were observed as DOM in the leachates increased. Another study using disturbed soil columns and field lysimeters was conducted under laboratory and field conditions to determine the leaching potential of eight commonly used pesticides during pepper cultivation [131]. Two soils with different organic matter (OM) content (soils A and B) were used for this purpose. Soil B, which had a lower OM content, was amended with compost (sheep manure). Endosulfan sulfate, tolclofos-methyl and malathion were found in the leachates of unamended soil B. However, pesticide residues were not detected in leachates from soil A (higher OM content) and amended soil B. A study was conducted to examine the effects of four different organic materials (composted pine bark, composted sheep manure, coir and spent coffee grounds) on the potential groundwater pollution of cadusafos (insecticide), pencycuron (fungicide), and propanil and isoxaben (herbicides) under laboratory conditions [132]. Adding OM to soil drastically reduced the movement of all pesticides. These results suggest the potential of using composted and agro-industrial waste to reduce groundwater pollution from pesticide leaching. Using the same OW, Fenoll et al. [133] evaluated the sorption, persistence, and mobility of eight symmetrical- and two asymmetrical-triazine herbicides. The results showed that *K*_OC_ increased in amended soils in all cases. According to the groundwater ubiquity score (GUS) index, all herbicides exhibited medium to high leachability in unamended soils. However, the addition of 10% (*w*/*w*) composted and agro-industrial waste strongly decreased the mobility of all herbicides.

Other authors examined the effects of two OAs (coir and composted sheep manure) on the sorption, persistence, and mobility of three pesticides, such as alachlor, chlorpyrifos and chlorfenvinphos [134]. The pesticides were found in the leachates of unamended soil, but in different proportions. Adding OA significantly increased the sorption of the studied pesticides. Consequently, their half-lives were longer in amended soil than in unamended soil. Notably, the amount recovered in leachates decreased in amended soils, except for chlorpyrifos, for which the recovery rate remained nearly unchanged. Pérez-Lucas et al. [64] studied the effects of adding composted sheep manure on the sorption, degradation, and leaching of four pesticides (myclobutanil, flonicamid, boscalid and penconazole) in an agricultural soil from a semi-arid region in southeastern Spain. The results of the experiments showed that the sorption capacity of the amended soil increased substantially in all cases. Rapid degradation of flonicamide was observed in both unamended and amended soils. In contrast, boscalid, myclobutanil, and penconazole exhibited high persistence (half-life greater than 110 days), though their half-lives were higher in amended soil due to increased adsorption. The leaching experiment showed that adding composted sheep manure greatly reduced the downward movement of pesticides through soil columns, particularly for penconazole and boscalid. Also, Pérez-Lucas et al. [135] evaluated the impact of three types of composted organic amendments (agroforestry, animal manure and agro-industrial) on the leachability of eight persistent herbicides. They concluded that adding OA increased herbicide sorption weakly but significantly in some cases and decreased persistence, with some exceptions, probably due to increased microbial activity. The findings show that adding OA to soil columns significantly increases retention and decreases herbicide leaching, which reduces the pesticide load in groundwater resources used for drinking water production.

### 5.3. Biobeds Systems

**Figure 5:**
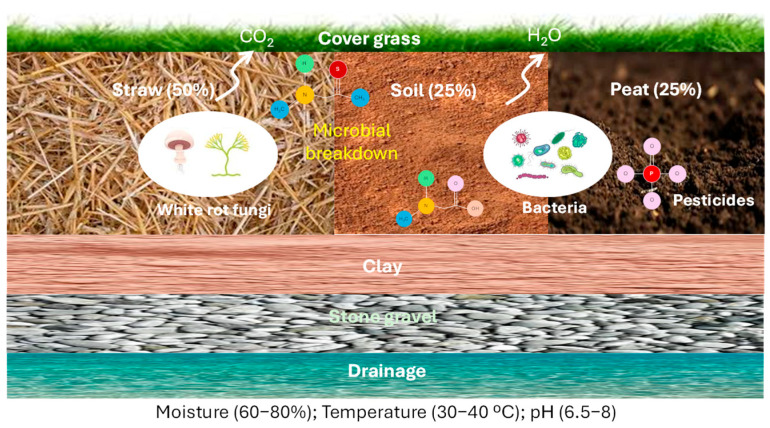
Typical biobed system.

Researchers have proposed biobeds (BBs) as low-cost, realistic, and effective alternatives for mitigating localized pesticide pollution, mainly in aqueous effluents but also in soil and air [38,39,136,137]. First described in Sweden in the 1990s by Torstenson and Castillo [138] as trenches filled with a mixture of 25% soil, 25% peat, and 50% wheat straw, and covered with a layer of grass, BBs are known as biomixtures (BMs) (Figure 5).

From this moment on, several EU countries have integrated BB systems, and experimental devices have been established in many areas around the world to control pesticide pollution [41]. The main factors contributing to the effectiveness of BBs to degrade pesticides are as follows: (i) BM composition, (ii) microorganisms, and (iii) physicochemical parameters of the BB system [139].

Wheat straw acts as an adsorbent for pesticides, provides physical support for the development of microbial communities, and offers essential nutrients for the growth of fungi and bacteria. It stimulates the production of ligninolytic enzymes, such as peroxidases and laccases, that are highly efficient at degrading pesticides. Peat, on the other hand, is a porous material that improves pesticide retention in the BB system. It also reduces the pH and regulates soil moisture, favoring pesticide dissipation. Finally, soil contributes by supplying microorganisms and stimulating microbial activity that mediates pesticide degradation [140]. The BB design has been extensively modified for two reasons. First, it considers the climate of the areas in which it is located. Second, it considers the accessibility of materials for the BM. Some examples of materials used instead of wheat straw include different other lignocellulosic materials, such as crop residues, orange peels, olive leaves, corn cobs, grape stalks, pine sawdust, barley husk, oat husk, sawdust or spent mushroom substrate, and compost or vermicompost in place of peat, among others [39,136,137,141,142,143,144,145,146,147,148,149].

In BB systems, the BM adsorbs pesticide residues and supports the growth of various microorganisms, including bacteria and fungi. These microorganisms are necessary for breaking down pesticides within the system. BBs are effective because of the BM’s high pesticide retention and the microorganism’s degradation potential. They use carbon, nitrogen, and phosphorus from pesticides as energy sources for their growth. The presence of lignocellulosic materials in BB has been shown to reduce the pH of the system, thereby creating an environment conducive to the proliferation of lignin-degrading fungi, including various species of white-rot fungi [150]. The production of extracellular enzymes by fungi, such as laccases, peroxidases and cytochrome P_450_, has been linked to the degradation of different pesticides [151]. The presence of peat in BM improves the development of white-rot fungi in BB. However, in BM without peat, the bacterial community is the main mediator of pesticide degradation. Bacteria may work together with fungi to speed up the breakdown of pesticides and the compounds that result from them. They can also produce different enzymes that break down pesticides. Some of these enzymes include dehalogenases, hydrolases, oxidoreductases, oxygenases, and esterases [152,153,154]. A metagenomics approach was used by Bergsveinson et al. [155] to assess the bacterial and fungal diversity in four BB systems used to treat rinsates that included different pesticides and concentrations. The study identified an average of 285 fungal genera and around 440 bacterial genera in each BB system. The efficient treatment of pesticide residues in BB systems relies on a great diversity of microorganisms. However, complete degradation of pesticides is not always ensured by the metabolic activities of the indigenous microbiota. For this reason, bioaugmentation strategies have been used. These strategies enhance the efficiency of pesticide biodegradation in BB systems, involving the addition of certain microorganisms, such as specific fungal and bacterial strains or microbial consortia that are characterized or non-characterized [156,157]. The main types of microorganisms added are white-rot fungi (*Trametes versicolor* and *Stereum hirsutum*) and Proteobacteria (Achromobacter, Bordetella, Pseudomonas, and Variovorax). These microorganisms are essential for breaking down pesticides in BB systems.

Other key parameters that differ from the composition of the BB system and the metabolic activity of microorganisms include temperature and moisture [158], incubation time [159], and pesticide concentration [160]. Studies that were reviewed indicate that, for the most efficient pesticide treatment, these parameters must be optimized. The review published by Mussali-Galante et al. [137] contains extensive data on the degradation of pesticides (insecticides, fungicides, and herbicides) in different BMs under different conditions.

The effectiveness of different types of SMS as pesticide adsorbents when used as BM has also been tested by some authors. Karas et al. [161] evaluated the adsorption and dissipation of different pesticides (imazalil, thiabendazole, ortho-phenylphenol, ethoxyquin, and diphenylamine) in BBs filled with BM composed of soil, straw, and SMS (*Pleurotus ostreatus*). The results show that all of the tested pesticides had higher adsorption and dissipation capacities when SMS-BM, especially SMS/straw/soil (50/25/25), was used compared to soil. The SMS from *Pleurotus ostreatus* cultivation showed great potential for retaining and dissipating fungicides such as imazalil and ortho-phenylphenol, either alone or mixed with straw and soil. The amount of fungicide leaching went down to less than 1% of the original amount in the wastewater from citrus fruit-packaging plants [162]. These results demonstrate the purification capacity of BB containing SMS-rich substrates that receive polluted effluents. Gao et al. [144] examined how three BM containing different SMS (*Pleurotus eryngii*, *Flammulina velutipes*, and *Lentinula edodes*) affected the dissipation of imidacloprid and chlorothalonil, concluding that SMS-BM could be used as an alternative to peat in the original BB design. Another study driven by Karanasios et al. [163] investigated whether SMS could be a substitute for peat in the usual mixture used in a BB system. They used BM of SMS, soil, and straw (1:1:2) to assess the degradation of eight pesticides (dimethoate, indoxacarb, metribuzin, terbuthylazine, buprofezin, azoxystrobin, metalaxyl, and iprodione) at two dosage rates. For instance, an increase in the adsorption of some pesticides such as terbuthylazine, metribuzin, metalaxyl, and indoxacarb using SMS (composted *Agaricus bisporus*) BM was observed as compared to soil, with the *K*_f_ values increased by between 1.3 and 7.7 times. Moreover, the findings indicated that the desorption percentages were less than 30% of the adsorbed amount for all the pesticides. In some cases, low desorption percentages were recorded (<0.4% for indoxacarb and lower for terbuthylazine). The study revealed that the SMS-BM accelerated the breakdown of the pesticides, regardless of the dosage, more rapidly than the soil alone.

## 6. Conclusions

Excessive and sometimes uncontrolled application of pesticides to combat natural plant enemies, such as pests, diseases, and weeds, frequently results in environmental pollution of environmental compartments (soil, water, air, and living organisms). Therefore, it is necessary to use remediation methods that can eliminate or at least reduce the pollution level caused by pesticides in the environment. According to the reviewed scientific literature, using waste from agricultural crops (crop waste, animal manure) and agri-food products (spent mushroom substrate, coir, spent coffee grounds, pine sawdust, etc.) as organic amendments is an effective method for retaining and degrading the residues of these compounds in the soil environment. This method also reduces their bioavailability and capacity to contaminate groundwater by leaching through the soil horizons. These materials can be used fresh after a drying and shredding process or composted to stabilize their organic matter content. Additionally, converting plant-based biomass into biochar and incorporating it into biobeds is an effective way to reduce pesticide pollution in soil. Therefore, using agri-food products can be considered a sustainable method. It provides an outlet for the enormous amount of agricultural and agro-industrial waste generated daily worldwide and contributes to the circular economy. Additionally, it reduces soil and groundwater pollution caused by the excessive use of pesticides.

The choice of the most appropriate method will depend, in each case, on the available waste, the necessary infrastructure for obtaining biochar or composted materials, the type of soil, climatic conditions, and the physicochemical characteristics of each pesticide. Furthermore, expanding these studies is strongly recommended to increase the value of agro-industrial waste and provide data on the behavior and fate of new pesticides.

## Figures and Tables

**Table 1:** Summary of the main processes, mechanisms and factors governing pesticide behavior and fate in the soil.

Process	Type	Mechanism	Main Parameters *	Factors
Adsorption	Physisorption	Van der Waals interactions	*K*_d_ = *C*_a_/*C*_e_ *K*_OC_ = (*K*_d_/OC) • 100 *q = K*_f_ • *C*^1/*n*^	Soil (pH, moisture, temperature, clay, and OM content) Pesticide (*K*_OW_, p*k*_a_, etc.)
	Chemisorption	Chemical binding		
Degradation	Photodegradation	Direct and indirect photolysis	*C*_t_ = *C*_0_ e^−kt^ DT_x_ = ln [100/100 − X]/*k*	UV light, photocatalysts, adsorption rate, soil pH, aeration, temperature and moisture, nutrients, microorganism type, and pesticide properties
	Biodegradation	Microorganisms’ breakdown		
	Chemical degradation	Oxidation, hydrolysis, etc.		
Leaching	Downward and interflow movement	Matrix and preferential flow	*K_OW_*, *K*_OC_ (leaching indices such as GUS, LIX, etc.)	Physicochemical properties of pesticide and soil, and mainly adsorption rate

* K_d_: distribution coefficient; *C*_a_: amount of pesticide adsorbed per unit mass of the adsorbent (M/M); *C*_e_: concentration of pesticide in solution (M/V); *K*_OC_: organic carbon–water partition coefficient (V/M); *K*_f_: Freundlich equilibrium constant; k: rate constant; *K*_OW_: octanol–water partition coefficient; DT_x_: disappearance time to remove a percentage (X) of the pesticide.

**Table 2:** Typical crop waste and livestock residues used as organic amendments to reduce soil pesticide pollution.

Organic Waste	Group	Source Materials
Grain crops	Cereal crops	Rice
		Corn
		Wheat
	Legume crops	Soybean
		Pea
	Tuber crops	Potato
		Cassava
Cash crops	Fiber crops	Cotton
		Hem and jute
	Oil crops	Rapeseed/canola
		Peanut/groundnut
		Sunflower
	Sugar crops	Sugarcane
		Sugar beets
	Beverage crops	Coffee
		Tea
	Medicinal crops	Herbs and plants (peppermint, lavender, calendula, etc.) Root and Shrub (ashwagandha, senna, shatavari, etc.) Medicinal trees (eucalyptus, neem, and stevia)
	Others	Walnut and chestnut
Fruit crops	Fruit peels and shells	
	Fruit seeds and stones	
	Fruit leaves, stems, and branches	
Livestock residues	Pig manure	
	Chicken manure/egg shells	
	Cow dung and dairy manure	
	Silkworm feces	

**Table 4:** Effect of biochar on the decrease in different pesticides in soils (Adapted from Losacco et al. [109]).

Raw Materials	Pesticide *	Decrease (%)
Cotton straw	Chlorpyrifos (I) Fipronil (I)	19 48
Maize straw and pig manure	Thiacloprid (I)	81
Rice husk	Methamidophos (I), phorate (I), isocarbophos (I), terbufos (I), malathion (I), parathion (I)	32
Pine woodchips	Pyrimethanil (F)	72
Red gum, woodchips	Pyrimethanil (F)	14
Oil palm and rice husk	Imazapyr (H)	70
Pine chip	Atrazine (H)	52
Cassava wastes	Atrazine (H)	96
Biochar mix	Atrazine (H)	90
Wheat straw	Pyrazosulfuron-ethyl (H)	47
Calligonum comosum	Atrazine (H)	45
Vegetable waste	Diuron (H)	45
Rice husk	Glyphosate (H)	82

*: I: insecticide; F: fungicide; and H: herbicide.

## Data Availability

No new data were created or analyzed in this study. Data sharing is not applicable to this article.

## Abbreviations

The following abbreviations are used in this manuscript:

AMPA	Aminomethylphosphonic acid
BB	Biobed
BC	Biochar
BM	Biomixture
DOC	Dissolved organic carbon
EU	European Union
GHG	Greenhouse gas
GUS	Groundwater Ubiquity Score
OA	Organic amendment
OC	Organic carbon
OM	Organic matter
ORs	Organic residues
OW	Organic waste
SMS	Spent mushroom substrate
SOM	Soil organic matter
WoS	Web of Science
